# protaTETHER – a method for the incorporation of variable linkers in protein fusions reveals impacts of linker flexibility in a PKAc‐GFP fusion protein

**DOI:** 10.1002/2211-5463.12414

**Published:** 2018-04-25

**Authors:** Jessica L. Norris, Robert M. Hughes

**Affiliations:** ^1^ Department of Chemistry East Carolina University Greenville NC USA

**Keywords:** linkers, protaTETHER, protein engineering, protein fusions, restriction enzyme‐free cloning

## Abstract

Protein fusions are of fundamental importance in the study of cellular biology and the elucidation of cell signaling pathways, and the importance of linkers for the proper function of protein fusions is well documented in the literature. However, there are few convenient methods available to experimentalists for the systematic implementation of linkers in protein fusions. In this work, we describe a universal approach to the creation and insertion of focused linker libraries into protein fusions. This process, deemed protaTETHER, utilizes reiterative oligomer design, PCR‐mediated linker library generation, and restriction enzyme‐free cloning methods in a straightforward, three‐step cloning process. We utilize a fusion between the catalytic subunit of cAMP‐dependent protein kinase A (PKAc) and green fluorescent protein (GFP) for the development of the protaTETHER method, implementing small linker libraries that vary by length, sequence, and predicted secondary structural elements. We analyze the impact of linker length and sequence on the expression, activity, and subcellular localization of the PKAc‐GFP fusions, and use these results to select a PKAc‐GFP fusion construct with robust expression and enzymatic activity. Based upon the results of both biochemical experiments and molecular modeling, we determine that linker flexibility is more important than linker length for optimal kinase activity and expression.

**Abbreviations**
FP: fluorescent protein
GFP: green fluorescent protein
PKAc: protein kinase A catalytic subunit
POI: protein of interest
VLR: variable linker region

Protein fusions are a mainstay of the biochemical toolkit, and the design and engineering of multifunctional protein fusions have long been a focus of the scientific community 1, 2. In particular, with the introduction of fluorescent proteins (FP) as reagents for both *in vitro* and *in vivo* studies, fusions between proteins of biochemical interest (POIs) and FPs have become standard methods for tracking both subcellular localization and activity via microscopy and immunofluorescence methods 3. More recently, applications of optogenetic proteins require the utilization of complex protein fusions that incorporate both FP and POI, and light‐sensitive components to achieve the desired biochemical activity, light responsivity, and imaging properties 4, 5. Utilizing current cloning methods and commercially available vectors, the assembly of DNA for the production of protein fusions can be both rapid and straightforward 6, 7. However, despite the relative ease of assembling genes for use in cell and animal studies, researchers often encounter numerous roadblocks when expressing protein fusions, including low activity levels, poor expression or solubility, protein degradation, aberrant activities, and off‐target subcellar localization 1. Many of these challenges stem from the design or absence of linker sequences between individual proteins in fusions 8. Indeed, numerous published studies of linker properties emphasize the importance of linker length, flexibility, and amino acid composition for the production of properly folded and active proteins 9, 10, 11, 12, 13, 14, 15, while others emphasize the impact(s) of the order of proteins in fusions on activity 16, 17. And while significant attention has been paid in the literature to the importance of linkers for fusions function, numerous studies that rely upon fusions do not report investigations of linker design, selection, or optimization. This potential oversight is likely driven by the current challenge of cloning and evaluating multiple versions of protein fusions for optimal activity, as currently available methods for linker introduction may be perceived as too specialized or overly cumbersome by experimentalists, and are thus not routinely implemented. In response to this experimental gap, we have developed a facile cloning method, deemed protaTETHER, which incorporates recently reported restriction enzyme‐free cloning methods with reiterative oligomer design, to enable the creation and introduction of both sequence‐ and structure‐focused linker libraries into protein fusions in a three‐step process. To demonstrate the application of protaTETHER for the optimization of protein fusions, we explore the response of a protein kinase A catalytic subunit (PKAc)‐GFP fusion to the incorporation of linker libraries in terms of activity, expression level, and subcellular localization.

## Results and Discussion

### protaTETHER cloning strategy

**Figure 1:**
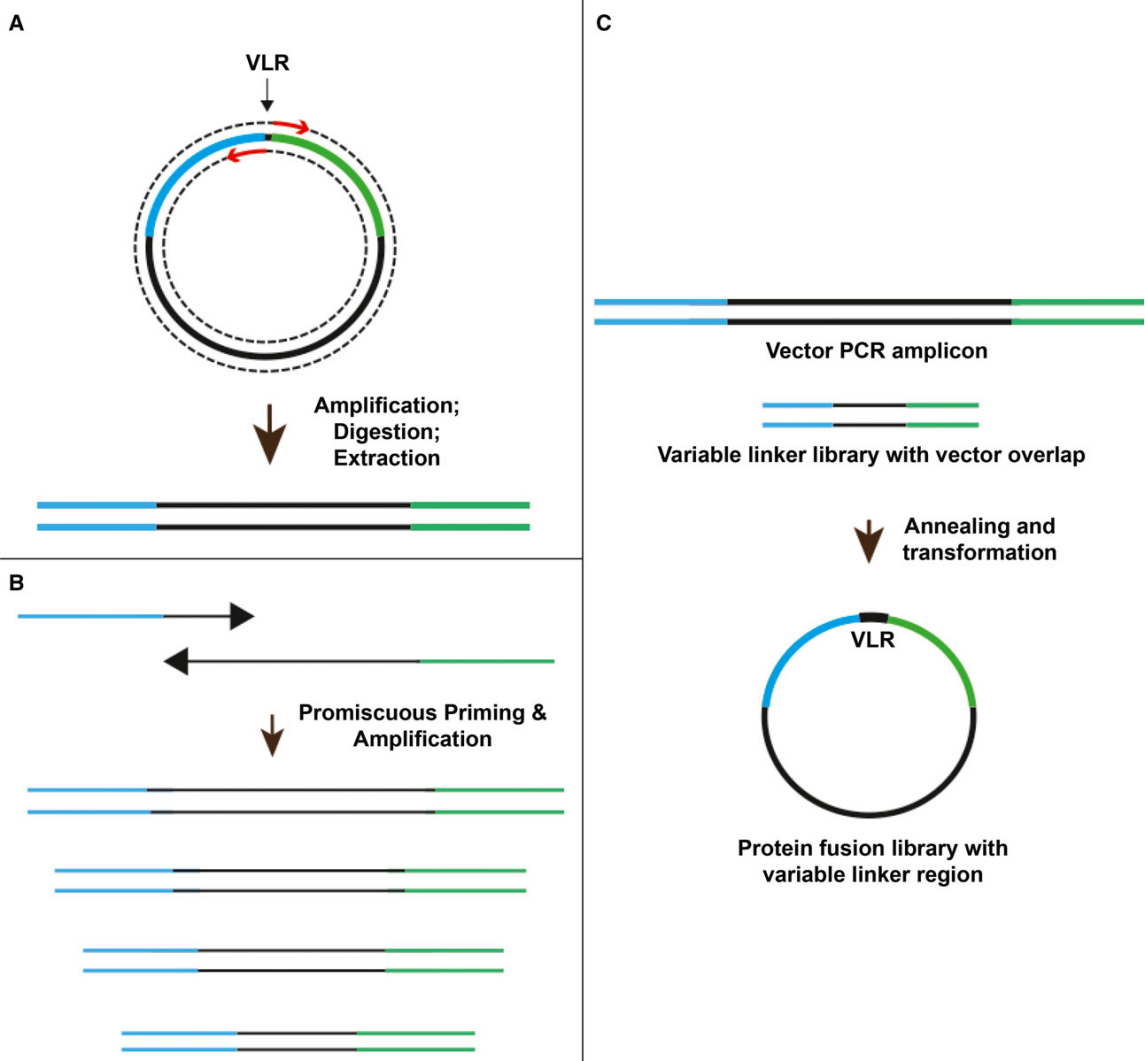
Overview of protaTETHER cloning strategy. (A) Primers for vector amplification (red arrows) are designed to amplify region immediately adjacent to variable linker region (VLR). Postamplification, the linear vector sequence is digested with DpnI and then gel extracted. PKAc gene indicated by blue lines; GFP gene indicated by green lines. (B) Oligomer sequences are designed to generate multiple amplicons via reiterative sequence design. The short forward primer contains 17 bp of overlap with the end of the PKAc gene in the target vector (blue) in addition to 1X or 2X linker repeat codons, while the longer reverse primer contains 17 bp of overlap with the start of the GFP in the target vector (green) in addition to 4 – 8X complimentary linker repeat codons. (C) Both vector (gel purified) and linker library (crude) amplicons are combined, briefly annealed at room temperature, and transformed into ultracompetent *E. coli*. The resulting plasmids are screened to identify constructs containing variable linker inserts.

Our cloning strategy combines a recently reported restriction enzyme‐free cloning method (Fast Cloning 18) with the design of PCR primers that contain both homology to the target vector region and reiterative linker sequences, thereby enabling multiple annealing sites during PCR amplification and the generation of linker libraries (Fig. 1). The cloning strategy was designed to circumvent purification steps that might impact the quality and/or sequence representation present in the PCR‐generated linker library. Restriction enzyme‐free cloning is an ideal solution to this problem and an effective means of skirting tedious purification steps 19. In the first step of the process, the target vector is amplified utilizing forward and reverse primers adjacent to the region targeted for linker insertion (variable linker region or VLR; Fig. 1A). The vector amplicon is then digested with DpnI to remove template DNA and further purified by gel extraction. In the second step (Fig. 1B), linker libraries are created from forward and reverse primers containing redundant linker sequences that provide multiple annealing sites for forward primers, which results in a diversity of PCR amplicons. These PCR products are carried forward without further purification. In the third and final step (Fig. 1C), the vector PCR product is combined with the variable linker library and, following a brief annealing step at room temperature, subsequently transformed into ultracompetent *E. coli*. Transformants are then isolated and characterized by Sanger sequencing. As our target protein fusion, we selected a mutant cAMP‐dependent protein kinase A catalytic subunit (PKAc), with the mutations H87Q and W196R, which have been demonstrated to prevent inhibition of catalytic activity by the PKA regulatory subunit 20, fused to a bright, photostable GFP encoded in a commercially available plasmid (phCMV‐GFP; Genlantis), to create a 607‐amino acid protein fusion (phCMV vector length including GFP: 4962 bp; PKA insert length: 1053 bp). PKA was an attractive target for illustrating the protaTETHER principle, due to its central role in numerous cell signaling pathways 21, 22, 23, as was GFP, due to its importance as a biomarker and its abundant use throughout the literature as a fusion protein 24, 25, 26.

### Development of the protaTETHER method

**Figure 2:**
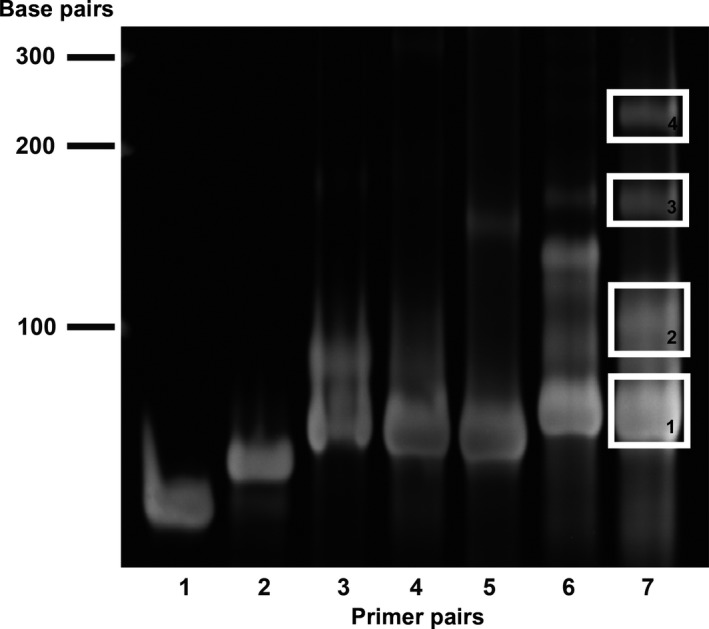
20% TBE/PAGE separation of GGS linker library reactions. Lanes 1‐7 correspond to Lines 1‐7 in Table 2. Bands indicated by white rectangles were excised, extracted, and cotransformed with target PKAc‐GFP DNA. Plasmid DNA from the resulting colonies was isolated and characterized by Sanger sequencing. Of the highlighted bands, transformation of DNA isolated from Band 1 resulted in PKAc‐VLR‐GFP constructs, while transformation of Bands 2 – 4 failed to produce PKAc‐VLR‐GFP constructs.

To develop a robust approach to the generation and cloning of linker libraries via the protaTETHER method, we selected the frequently utilized GGS amino acid motif. 1 We designed reverse primers that incorporated codons encoding one, two, three, or four GGS repeats, and forward primers that encoded one and two GGS repeats (Table 1). For our initial screen of primer pairs, we paired both GGS1X, GGS2X, and GGS3X forward and reverse primer pairs (**1, 2**, and **3**; Table 2) in addition to combinations of GGS1X forward primer with GGS3X and GGS4X reverse primers (**4** and **6**; Table 2) and GGS2X forward primer with GGS3X and GGS4X reverse primers (**5** and **7**; Table 2). We then cotransformed the resulting PCR amplicons and the target vector containing PKAc and GFP components into ultracompetent *E. coli*. PCR amplicons resulting from the pairing of 1X forward and reverse primers and amplicons resulting from the pairing of 2X forward and reverse primers failed to produce plasmids with linker inserts (Table 2). By contrast, amplicons derived from 3X forward and reverse primers gave PKAc‐GFP fusions with 2X and 3X GGS repeat sequences. Transformation of PCR amplicons resulting from combination of a 1X GGS forward primer with 3X or 4X GGS reverse primers gave similar results, producing only a limited subset of linkers. However, transformation of the amplicons resulting from combination of the 2X GGS forward primer (GGS2X_Fwd; Table 2) with a 4X reverse primer (GGS4X_Rev; Table 2) provided the best coverage of all possible linker sequences (1X, 2X, 3X, and 4X), generating linkers ranging from 3 amino acids (GGS) to 12 amino acids (GGSGGSGGSGGS; Table 2, **line 7**). Importantly, 9 of 12 colonies sequenced in this trial contained VLR inserts, resulting in a higher frequency of successful linker inserts than previously tested primer combinations. The superior combination of the 2X forward primer with the 4X reverse primer likely results from the stronger annealing achieved by the 2X forward/4X reverse overlap (*T*_m_ = 70 °C; salt‐adjusted) over the 1X forward/4X reverse overlap (*T*_m_ = 34 °C; salt‐adjusted), while the greater sequence coverage observed with the 2X/4X pair results from the additional sequence space encoded by the longer GGS4X reverse primer. Furthermore, the ability of the GGS2X_Fwd/GGS4X_Rev primer pair to produce the truncated GGS1X insert likely stems from annealing of the outermost GGS repeat sequence of the forward primer with the outermost GGS repeat sequence of the GGS4X reverse primer. We analyzed the amplicons produced by the different primer pairs via TBE/PAGE (Fig. 2), as efforts to resolve the small products on a high percentage agarose gel were unsuccessful. We were able to distinguish the amplicons produced by the primer pairs listed in Table 2 by size, and, in addition to the expected prominent lower molecular weight bands, we also observed the presence of numerous higher MW bands. To determine which of these amplicons could produce VLR inserts, we excised and extracted these bands and cotransformed the resulting purified PCR products with the target PKAc‐GFP sequence. Transformation of the most prominent band (Fig. 2; **Lane 7, Band 1**) produced colonies with VLR inserts, while transformation of the other isolated and extracted bands resulted in colonies with no inserts, indicating that the nonspecific, higher MW PCR products are unable to successfully anneal with the vector amplicon, thus precluding their use for the production of significantly longer linker sequences.

**Table 1:** Initial primers tested for incorporation of GGS linkers in the PKAc‐GFP protein fusion

Primer name	Sequence	Description
GFPNterm_Fwd	ATGGCTAGCAAAGGAGA	PKA‐GFP vector primer F; homology with GFP N‐term
PKACterm_Rev	AAACTCAGTAAACTCCT	PKA‐GFP vector primer R; homology with PKA C‐term
GGS1X_Fwd	*AGGAGTTTACTGAGTTT* GGCGGCAGC	Variable insert F primer, GGS; *17 bp homology with C‐term of PKAcs*
GGS1X_Rev	*TCTCCTTTGCTAGCCAT* GCTGCCGCC	Variable insert R primer; GGS; *17 bp homology with N‐term of GFP*
GGS2X_Fwd	*AGGAGTTTACTGAGTTT* (GGCGGCAGC)_2_	Variable insert F primer; GGS x 2; *17 bp homology with C‐term of PKAcs*
GGS2X_Rev	*TCTCCTTTGCTAGCCAT* (GCTGCCGCC)_2_	Variable insert R primer, GGS x 2; *17 bp homology with N‐term of GFP*
GGS3X_Fwd	*AGGAGTTTACTGAGTTT* (GGCGGCAGC)_3_	Variable insert F primer; GGS x 3; *17 bp homology with C‐term of PKAcs*
GGS3X_Rev	*TCTCCTTTGCTAGCCAT* (GCTGCCGCC)_3_	Variable insert R primer; GGS x 3; *17 bp homology with N‐term of GFP*
GGS4X_Rev	*TCTCCTTTGCTAGCCAT* (GCTGCCGCC)_4_	Variable insert R primer; GGS x 4; *17 bp homology with N‐term of GFP*

**Table 2:** Sequencing results for transformation of GGS primer pairs. Results indicate both linker length and, in bold parenthesis, frequency of occurrence

	Primer pair	Result	Colonies sequenced	Colonies with inserts
1	GGS1X_Fwd/GGS1X_Rev	No inserts	12	0
2	GGS2X_Fwd/GGS2X_Rev	No inserts	12	0
3	GGS3X_Fwd/GGS3X_Rev	2X(**4**) & 3X(**2**) GGS	12	6
4	GGS1X_Fwd/GGS3X_Rev	2X(**2**) & 3X(**4**) GGS	12	6
5	GGS2X_Fwd/GGS3X_Rev	3X(**4**) GGS	12	4
6	GGS1X_Fwd/GGS4X_Rev	2X(**1**) & 4X(**5**) GGS	12	6
7	GGS2X_Fwd/GGS4X_Rev	1X(**1**), 2X(**2**), 3X(**2**), 4X(**4**) GGS	12	9

### GGSD linker library cloning

Having identified primer pairs (2X forward primer/4X reverse primer) that facilitated variable linker insertion of the GGS motif via the protaTETHER method, we hypothesized that the production of libraries for the incorporation of significantly longer linkers could be addressed via the utilization of a significantly longer reverse primer. To explore this notion, we acquired a forward primer that encoded two GGSD repeats (another frequently employed flexible linker sequence 1) and paired it with a reverse primer encoding for eight GGSD repeats (Table 3). Annealing of the resulting amplicon with the PKAc‐GFP vector and transformation into ultracompetent cells resulted in plasmids that encoded for linkers ranging from GGSD3X (12 amino acids in length) repeats to GGSD10X (40 amino acids in length) repeats. In this experiment, 8 of 16 colonies sequenced contained variable GGSD linker inserts. The presence of GGSD9X and GGSD10X linkers indicated that multiple rounds of PCR amplification have the potential to produce linkers that exceed the length of the reverse primer template via promiscuous overlap of the forward primer with full‐length amplicons. As a result, if the production of even longer linkers is desired via protaTETHER, it may be possible to accomplish this by additional rounds of thermal cycling during the library generation step.

**Table 3:** Primers for incorporation of GGSD linkers in the PKAc‐GFP protein fusion

Primer name	Sequence	Description
GGSD_Fwd	*AGGAGTTTACTGAGTTT* GGCGGCAGCGACGGCGGC	Variable insert F primer; GGSDGG; *homology with C‐term of PKA*
GGSD8X_Rev	*TCTCCTTTGCTAGCCAT* (GTCGCTGCCGCC)_8_	Variable insert R primer; GGSD x 8; *17 bp homology with N‐term of GFP*

### Incorporation of structured linkers

Finally, having generated two different sets of PKAc‐GFP fusions containing variable length flexible linkers, we then generated additional linker libraries encoding for single or double amino acid repeats (poly L, poly K, poly KR) to implement structurally rigid (helical) linkers (Table 4), in addition to the generation of a flexible poly G linker, as control constructs. While coverage of sequence space was less robust for these experiments than for the GGS linker series (Table 5), we were able to isolate PKAc‐VLR‐GFP constructs containing poly Gly linker sequences ranging from 9X to 14X, poly Lys sequences ranging from 9X to 14X, and a subset of variants for the poly Leu and KR repeat linkers. As a comparison to our flexible GGS and GGSD linkers, we selected the 12X lysine linker (K12), the 6X KR repeat linker (KR6), the 10X leucine linker (L10), and the 12X glycine linker (G12).

**Table 4:** Initial primers for incorporation of polyG, polyK, polyKR, and polyL linkers in PKA‐GFP protein fusion

Primer name	Sequence	Description
polyG1X_Fwd	AGGAGTTTACTGAGTTT GGCGGCGGC	Variable insert F primer; glycine repeats; *17 bp homology with C‐term of PKAcs*
polyG2X_Fwd	AGGAGTTTACTGAGTTT (GGCGGCGGC)_2_	Variable insert F primer; glycine repeats; *17 bp homology with C‐term of PKAcs*
polyG12X_Rev	TCTCCTTTGCTAGCCAT (GCCGCCGCC)_4_	Variable insert R primer; glycine repeats; *17 bp homology with N‐term of GFP*
polyKR_Fwd	AGGAGTTTACTGAGTTT AAACGCAAA	Variable insert F primer; Lys‐Arg repeats; *17 bp homology with C‐term of PKAcs*
polyKR_Fwd2	AGGAGTTTACTGAGTTT (AAACGCAAA)_2_	Variable insert F primer; Lys‐Arg repeats; *17 bp homology with C‐term of PKAcs*
polyKR6X_Rev	TCTCCTTTGCTAGCCAT (GCGTTT)_6_	Variable insert R primer; Lys‐Arg repeats; *17 bp homology with N‐term of GFP*
polyK_Fwd	AGGAGTTTACTGAGTTT AAGAAGAAG	Variable insert F primer; lysine repeats; *17 bp homology with C‐term of PKAcs*
polyK_Fwd2	AGGAGTTTACTGAGTTT (AAGAAGAAG)_2_	Variable insert F primer; lysine repeats; *17 bp homology with C‐term of PKAcs*
polyK12X_Rev	TCTCCTTTGCTAGCCAT (CTTCTTCTT)_4_	Variable insert R primer; lysine repeats; *17 bp homology with N‐term of GFP*
polyL_Fwd	AGGAGTTTACTGAGTTT CTTCTTCTT	Variable insert F primer; leucine repeats; *17 bp homology with C‐term of PKAcs*
polyL_Fwd2	AGGAGTTTACTGAGTTT (CTTCTTCTT)_2_	Variable insert F primer; leucine repeats; *17 bp homology with C‐term of PKAcs*
polyL12X_Rev	TCTCCTTTGCTAGCCAT (AAGAAGAAG)_4_	Variable insert R primer; leucine repeats; *17 bp homology with N‐term of GFP*

**Table 5:** Sequencing results for transformation of polyG, polyK, polyKR, and polyL primer pairs. Results indicate both linker length and, in bold parenthesis, frequency of occurrence

	Primer pair	Result	Colonies sequenced	Colonies with inserts
1	polyG1X_Fwd/polyG12X_Rev	10X**(2),** 11X**(1**), 12X**(1**), 14X**(1)**Gly	14	5
2	polyG2X_Fwd/polyG12X_Rev	9X**(3)** and 11X**(1**)Gly	8	4
3	polyKR_Fwd/polyKR6X_Rev	6X**(3)** and 7X**(1)** KR	14	4
4	polyKR_Fwd2/polyKR6X_Rev	7X**(1)**KR	8	1
5	polyK_Fwd/polyK12X_Rev	9X**(1)**, 10X**(2)**, 11X**(1)**, 12X**(2)**, 13X**(1)**, 14X**(2)** Lys	14	9
6	polyK_Fwd2/polyK12X_Rev	8X**(1)** and 12X**(1)** Lys	8	2
7	polyL_Fwd/polyL12X_Rev	10X**(1)** Leu	14	1
8	polyL_Fwd2/polyL12X_Rev	8X**(1)** and 13X**(1)** Leu	8	2

### Evaluation of PKAc‐VLR‐GFP fusions expression

**Figure 3:**
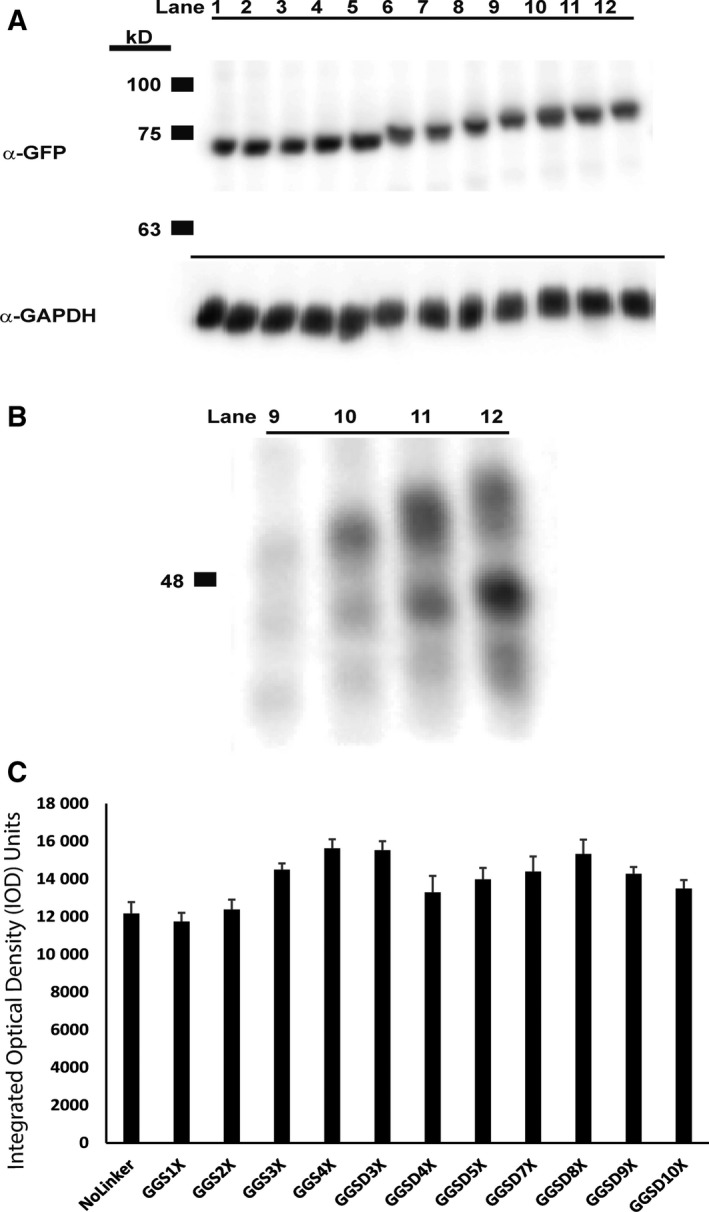
(A) Western Blot (α‐GFP) of PKA‐VLR‐GFP‐transfected HeLa cell lysates. VLR (Lanes 1 – 12) are as follows: no linker; GGS1X; GGS2X; GGS3X; GGS4X; GGSD3X; GGSD4X; GGSD5X; GGSD7X; GGSD8X; GGSD9X; GGSD10X. GAPDH was used as a loading control. (B) Immunopositive α‐GFP degradation bands are visible in lanes 9 – 12 (GGSD7X; GGSD8X; GGSD9X; GGSD10X). C. Total expression of full‐length protein was quantified by densitometry analysis in ImageJ.

**Figure 4:**
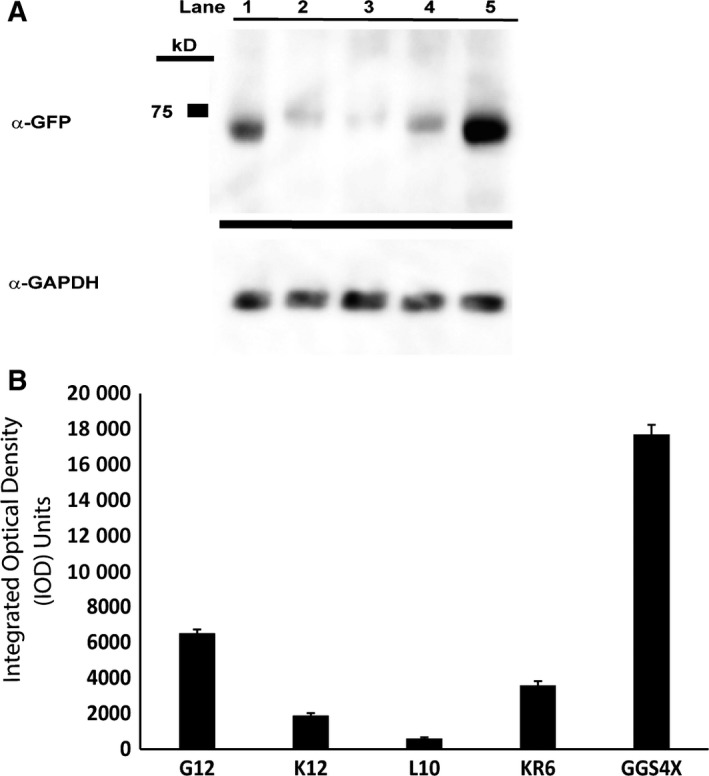
Expression of PKAc‐VLR‐GFP constructs containing G12, K12, L10, and KR6 linkers. (A) Western blot (α‐GFP (upper); α‐GAPDH (lower)) of PKAc‐VLR‐GFP chimera expressed in HeLa cells. (B) Densitometry analysis and comparison to PKAc‐GGS4X‐GFP expression. An average of three densitometry measurements per sample was compiled in FIJI (ImageJ) and normalized to GAPDH expression.

**Figure 5:**
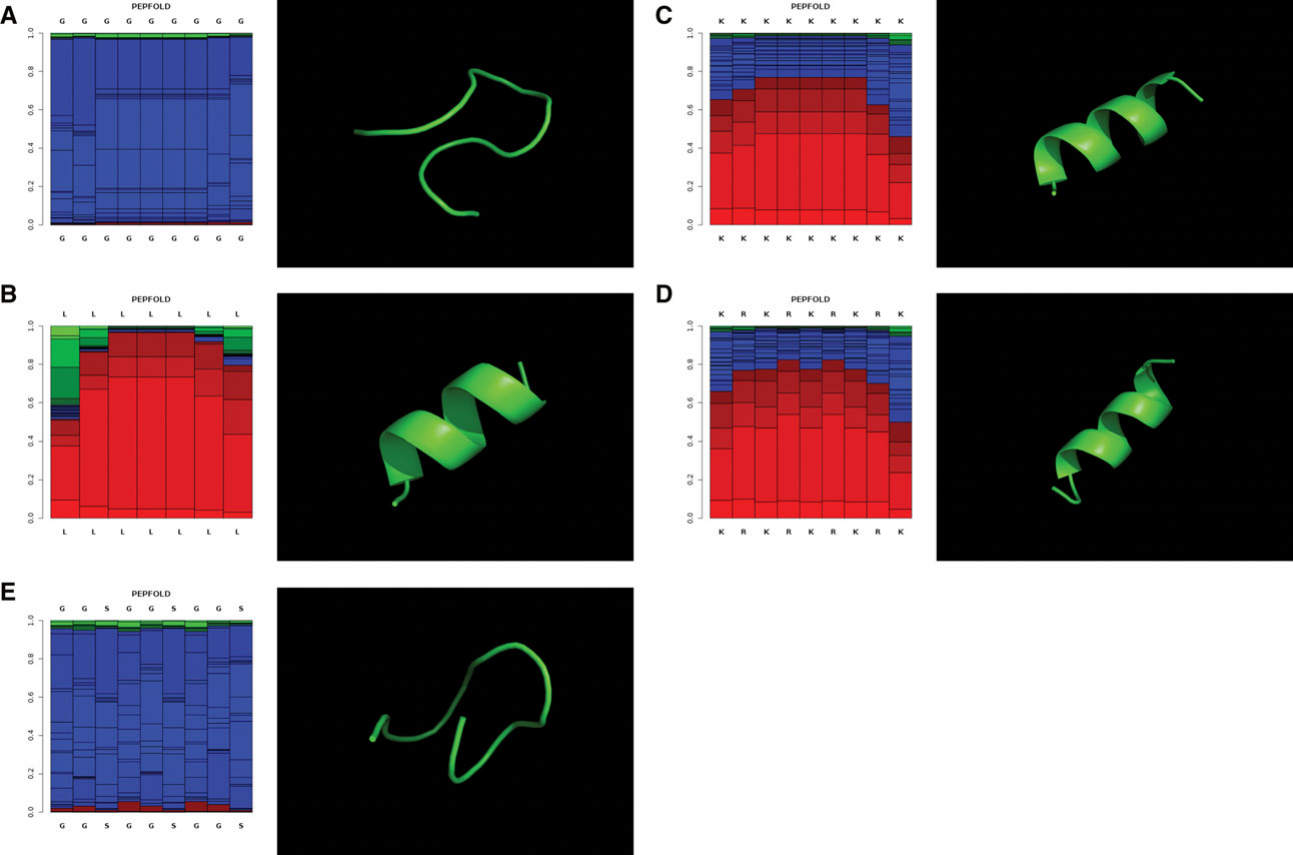
PEPFOLD 2.0 calculations of peptide secondary structural content and ribbon drawings of predicted structures. (A) G12 peptide; (B) L10 peptide; (C) K12 peptide; (D) KR6 peptide; (E) GGS4X peptide. PEPFOLD plots show the local structure prediction profiles (color code: red: helical, green: extended, blue: coil) predict helical structures for K12, L10, and KR6 peptides, and random coil structures for G12 and GGS4X peptides. Structural models depicted with green ribbon diagrams (PyMOL) are the top structures from 10 best structures generated by the PEPFOLD 2.0 structural prediction server.

Upon creation of a series of PKAc‐VLR‐GFP fusions, we next sought to investigate the impact of the introduced linkers on protein expression, activity, and localization. We expressed our constructs in HeLa cells by transient transfection and initially characterized protein expression levels by western blot (Fig. 3). Via western blot, we observed robust expression of the no‐linker control fusion, the GGS linker series, and the GGSD linker series. Constructs with longer linkers (7X and higher) in the GGSD series were accompanied by lower MW degradation bands (Fig. 3B), indicating that linkers beyond 20 amino acids in length may render fusions susceptible to proteolytic degradation, potentially making these long linkers undesirable under certain circumstances. In addition to the GGS and GGSD fusions, we also expressed PKAc‐VLR‐GFP fusions containing the structured linkers K12, L10, and KR6. Interestingly, these constructs expressed at lower levels than their no linker, GGS, GGSD, and G12 counterparts (Fig. 4). This is likely due to structural rigidity imparted by the polyL, polyK, and polyKR linkers, which potentially interfere with protein folding and thus result in low protein expression. 27 This possibility was confirmed via structural calculations (PEPFOLD 2.0 28, 29), which predicted alpha‐helical structures for L12, K12, and KR6 linkers, and random coil structures for the G12 and GGS4X peptide sequences (Fig. 5). Structurally rigid linkers, which are important for domain separation and activity in a number of protein fusions 1, are clearly suboptimal in the context of this particular PKAc‐GFP fusion by reducing protein expression. There is also another possible scenario, in which the presence of multiple codon repeat sequences impedes translation efficiency where the supply of charged tRNA is rate limiting, resulting in lower overall expression levels for highly redundant amino acid sequences such as G12, K12, and L10 30. We will further explore this notion in future studies of single amino acid linker sequences.

### Evaluation of PKAc‐VLR‐GFP fusion enzymatic activity

**Figure 6:**
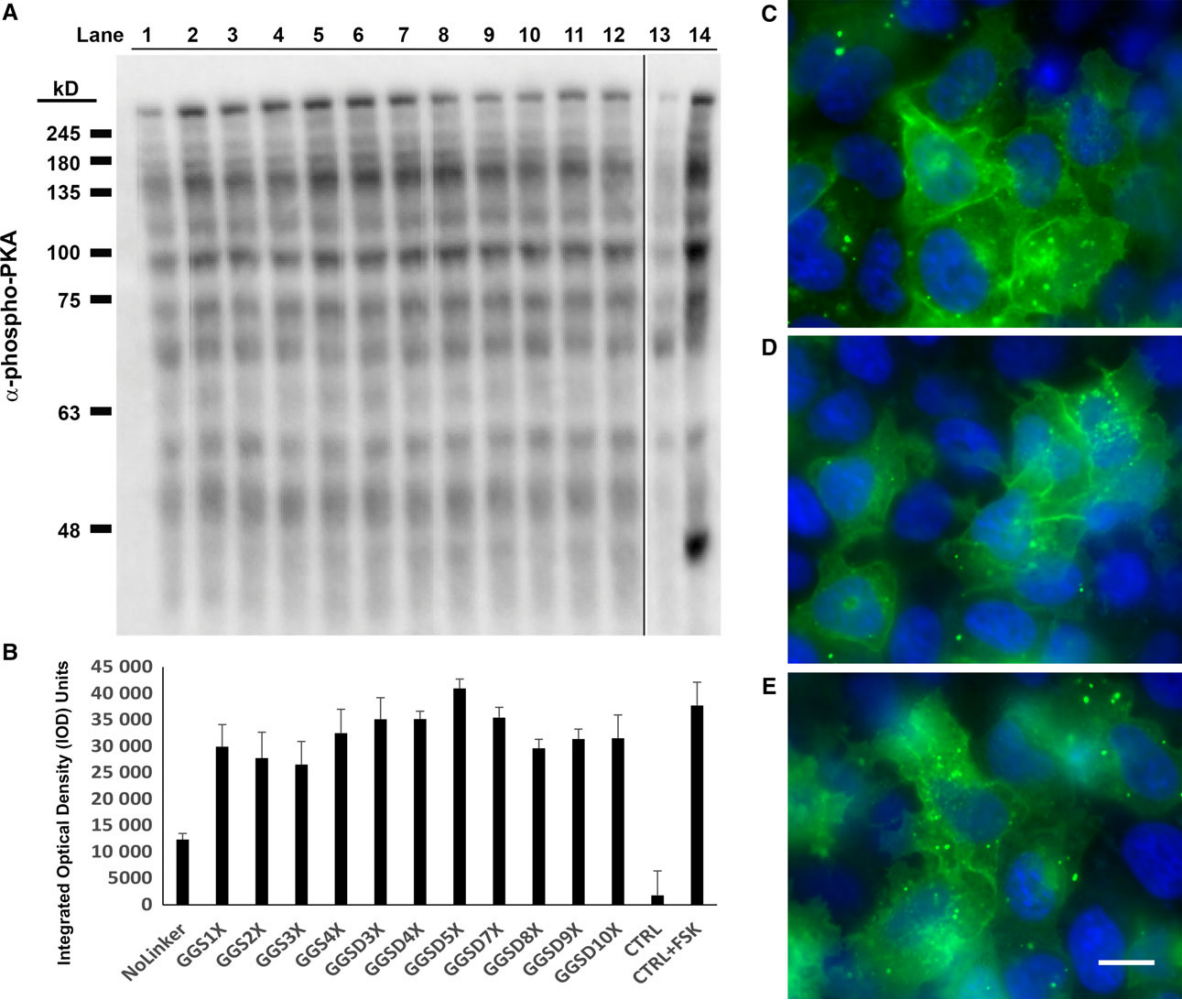
(A) Western Blot (α‐PKA phosphosubstrate) of PKA‐VLR‐GFP‐transfected HeLa cell lysates. VLR (Lanes 1 – 14) are as follows: no linker; GGS1X; GGS2X; GGS3X; GGS4X; GGSD3X; GGSD4X; GGSD5X; GGSD7X; GGSD8X; GGSD9X; GGSD10X; no transfect control; Forskolin‐treated control. GAPDH was used as a loading control. (B) Band intensities were quantified by densitometry analysis in ImageJ. Identical rectangular selection areas (ranging from the dark bands above 245‐kD MW marker down to the second faint band below the 48 kD MW marker) were used to measure the total optical density of each lane. Error bars represent the average of three replicates for each condition. (C–E) No linker, GGSD, and GGS iterations of PKA‐VLR‐GFP constructs are membrane localized. Images shown are (C) PKA‐GGSD3X‐GFP, (D) PKA‐GGS4X‐GFP, (E) PKA‐GFP. Confocal microscopy images (Olympus IX2‐DSU Tandem Spinning Disk Confocal; 60X objective) were acquired using eGFP and DAPI filter cubes. Channel overlays created with FIJI (ImageJ). Scale bar = 10 microns.

**Figure 7:**
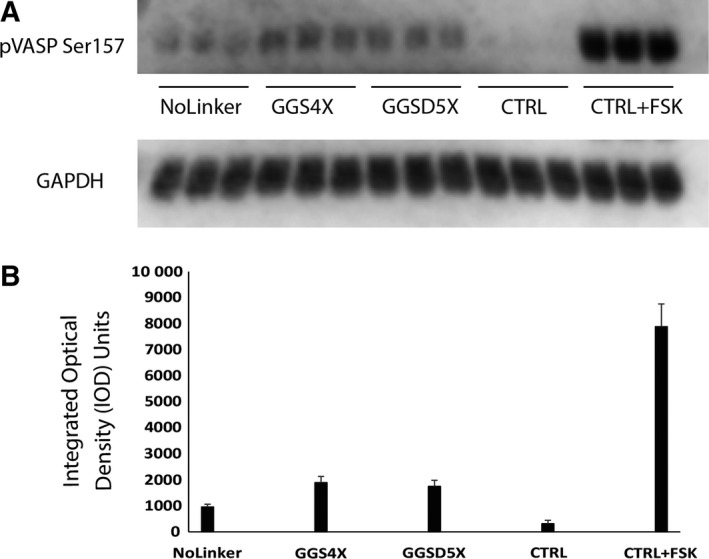
(A) phospho‐VASP (Ser157) western Blot of indicated PKAc‐VLR‐GFP chimera and (B) western Blot densitometry. An average of three technical replicates per sample was compiled in FIJI (ImageJ) and normalized to GAPDH expression.

**Figure 8:**
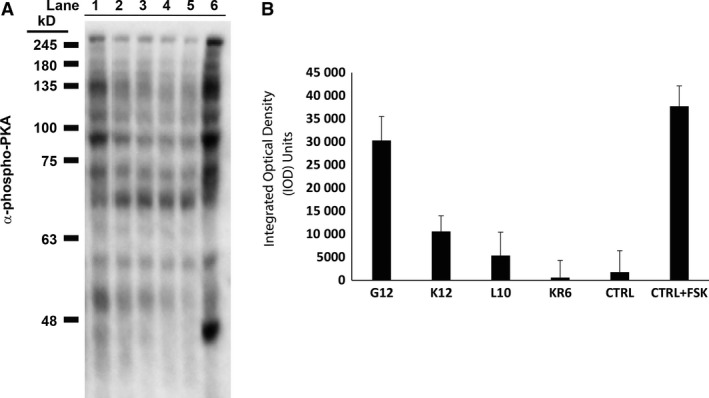
PKA substrate phosphorylation level of polyG, polyL, polyK, and poly KR linker constructs. (A) Global PKA‐dependent phosphorylation in PKAc‐VLR‐GFP‐transfected HeLa cells assessed with phospho‐PKA substrate antibody. Lane 1: G12 linker; Lane 2: K12 linker; Lane 3: L10 linker; Lane 4: KR6 linker; Lane 5: nontransfected control cells; Lane 6: forskolin‐treated nontransfected control cells. (B) Western blot densitometry measurements. An average of three densitometry measurements per sample was compiled in FIJI (ImageJ) and normalized to GAPDH expression.

After confirmation of expression of full‐length PKAc‐VLR‐GFP fusions, we then sought to investigate the influence of linker sequence upon PKAc activity. Utilizing a phospho‐PKA substrate antibody, we first analyzed global PKAc activity by western blot (Fig. 6). In general, we found that the abundance of PKA‐dependent phosphorylation increased with the incorporation of flexible linkers, regardless of length. In the GGS linker series, for example, addition of flexible linker sequence increased global PKA substrate phosphorylation (Fig. 6B), with the largest increase in observed activity occurring with the introduction of a GGS1X linker (a 2.4‐fold increase in total phosphorylation versus the no‐linker control; *P* < 0.001; one‐way ANOVA; Holm–Sidak Method), followed by a statistically insignificant increase in activity with the introduction of the 12‐amino acid‐long GGS4X linker (*P* = 1.000; GGS1X versus GGS4X; one‐way ANOVA; Holm–Sidak Method). In the GGSD linker series, we profiled linkers ranging from GGSD3X to GGSD 10X, and found that the PKA‐dependent phosphorylation peaked with the 20‐amino acid GGSD linker (GGSD5X; a 3.3‐fold increase in total phosphorylation over the no‐linker control) before decreasing slightly with the incorporation of longer linkers, although the difference between the GGSD5X linker and the GGS1X linker was found to be statistically insignificant (*P* = 0.078; one‐way ANOVA; Holm–Sidak Method). Notably, the PKA‐dependent phosphorylation levels observed with the GGSD5X linker are statistically similar to those resulting from cells treated with the potent adenylate cyclase activator forskolin (*P* = 1.000; one‐way ANOVA; Holm–Sidak Method). In addition to exploring global PKA‐dependent phosphorylation levels, we also examined PKA‐dependent phosphorylation of vasodilator‐stimulated phosphoprotein (VASP) at a known PKA phosphorylation site (Serine 157; Fig. 7), and found that VASP phosphorylation levels corroborated the global trends observed with the phospho‐PKA substrate antibody for the no linker, GGS4X, and GGSD5X linker constructs, where the incorporation of a flexible linker resulted in higher global kinase activity regardless of its identity. By comparison, in the structurally rigid linker sequences that we examined (K12, L10, and KR6), total PKA‐dependent activity fell dramatically, correlating with the previously observed trends in protein expression (Fig. 8). While the flexible G12 linker sequence exhibited expression and activity comparable to the GGS/GGSD series linkers, constructs with structurally rigid linkers exhibited PKA‐dependent phosphorylation at or near baseline levels, effectively demonstrating the importance of linker flexibility on both expression and activity for the PKAc‐GFP fusion. Taking these results in sum, the 3‐amino acid GGS1X linker was sufficient to enhance the observed kinase activity over the no‐linker PKAc‐GFP control, while the ‘optimal’ GGSD5X linker (20 amino acids) gave a statistically insignificant increase over the GGS1X sequence By contrast, structured linkers with up to 3X the sequence length of the three‐amino acid GGS sequence hindered both expression and, by extension, observed activity.

### Evaluation of PKAc‐VLR‐GFP fusions subcellular localization

**Figure 9:**
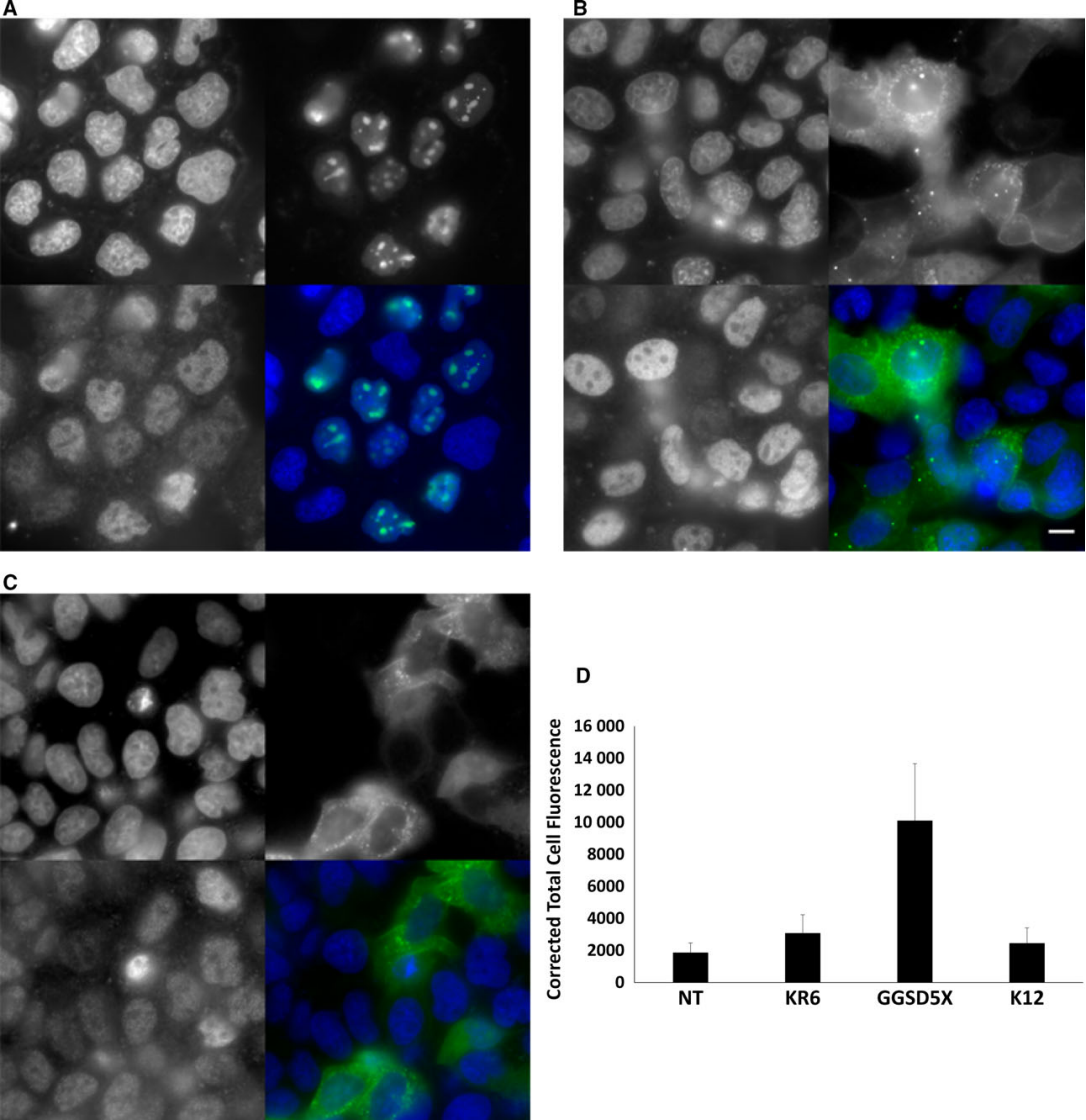
(A) KR6, (B) GGSD5X, and (C) K12 iterations of PKA‐VLR‐GFP constructs were fixed and immunostained for phospho‐CREB/phospho‐AIF. The four panels in each image (A–C) show (clockwise, starting from lower left): pCreb/pAIF immunofluorescence; Hoechst 33342; eGFP; overlay of Hoechst 33342 and eGFP. Scale bar = 10 microns. (D) corrected total cell fluorescence measurements of immunostained nuclei of PKA‐VLR‐GFP‐transfected HeLa cells (*n* = 10 cells per construct).

Subsequent to our investigations of linker effects on expression and activity, we sought to analyze the impact of linker sequence upon the subcellular localization of PKAc‐VLR‐GFP fusions. PKAc possesses an N‐terminal myristoylation sequence, which is post‐translationally modified to render the protein membrane bound 31. We observed this anticipated localization pattern in transfected HeLa cells expressing PKAc‐GFP fusions containing no linker, the flexible GGS linker series, and the flexible GGSD linker series (Fig. 6C–E). We suspected that the structured linker sequences (K12, KR6, and L10), which negatively impacted expression and activity, might also significantly alter protein localization, in particular those containing highly charged linker sequences. Upon analysis of our polybasic linkers, we found that while the polyK linker (K12) and hydrophobic L10 did not significantly impact protein localization (they remain membrane bound), the presence of KR linker repeats (KR6) resulted in the sequestration of the PKAc‐VLR‐GFP construct to nucleosomes (Fig. 9). This is consistent with previously reported peptides containing KR repeats, which are primarily nuclear localized, but does not explain the non‐nuclear distribution of the K10 linker sequence, as Lys repeats can also induce nuclear localization. 32 Nonetheless, it is significant to note that seemingly innocuous changes in linker sequence can lead to vastly different subcellular localizations. It is therefore important to analyze not only expression and activity but also localization between even closely related linkers. Given these differences in subcellular distribution, we also examined the role of subcellular localization on PKA activity, probing whether the PKA‐dependent phosphorylation states of nuclear‐localized proteins (pCREB (Ser 133)/pATF (Ser 63)) were dependent upon the localization of our PKAc‐VLR‐GFP fusions. Subsequent immunostaining of cells transfected with the plasma membrane‐localized GGSD5X PKAc‐VLR‐GFP construct showed significantly higher nuclear phosphorylation levels of pCREB/pATF than nucleosome‐localized KR6 linker construct and the plasma membrane‐localized K12 linker construct (Fig. 9). This result is consistent with current models of PKA‐dependent CREB phosphorylation, in which upstream activation of cytoplasmic PKA results in downstream phosphorylation of ERK and nuclear localization of the ERK/RSK complex 33, and also suggests that the variation of linker sequence alone in protein fusions could be useful in exploring how both localization and activity of various enzymes impact selective targeting of cell signaling pathways.

## Conclusion

The implementation and evaluation of linker sequences in the construction of protein fusions are an important, yet often overlooked, aspect of protein design. By removing experimental barriers to linker design and optimization, it is possible that linker optimization might become a more routine component of studies which rely upon protein fusions as either reporters or inducers of biochemical activity. In this study, we designed a three‐step process for implementation of multiple linker sequences generated by a single PCR. From pools of linkers implemented in PKAc‐GFP fusions, we subsequently identified linkers that promoted high levels of expression and kinase activity, resulting in kinase‐dependent phosphorylation levels similar to treatment with a small molecule. The major conclusion from this work, however, is that the incorporation of a short flexible sequence gave a large, significant 2.6‐fold enhancement in observed kinase activity, while the incorporation of longer flexible linkers provided diminishing returns, and incorporation of structurally rigid linkers actually impeded protein activity and expression. Furthermore, the observed changes in subcellular localization between the KR6 fusion and the remaining PKAc‐VLR‐GFP constructs demonstrate the importance of analyzing the impacts that even small changes in linker sequence may have upon fusion localization. As a result of this work, we suggest that other researchers will benefit by incorporating the protaTETHER approach to the evaluation of linker effects in newly synthesized protein fusions, first by evaluating the impact of rigid versus flexible tether incorporation, followed by optimizing the length of the tether (flexible or rigid) that best enhances the desired activity. While the protaTETHER approach does not address the problem of *de novo* linker sequence design and optimization, it does enable rapid assessment of the most fundamental aspects of linker design (length and structural flexibility). As a result, we anticipate that the protaTETHER process will be of practical use to biochemists and molecular biologists who are involved in the design and characterization of protein fusions, and can be readily achieved by almost all laboratories possessing basic molecular biology equipment and access to a source of synthetic oligomers. This approach will also be of utility to those seeking to validate and/or optimize the protein fusions employed in numerous biochemical studies and, in addition, may find utility in the crystallography community, as it will enable iterative replacement and or modification of loops or random coil regions in proteins as a part of crystallography trials. Further studies implementing this method in the production of multifunctional enzyme fusions for biotechnical applications are currently underway in our laboratory.

## Materials and methods

### Cloning of PKAc‐GFP fusion

A nonregulatory subunit inhibited mutant (H87Q/W196R) of the catalytic subunit of cAMP‐dependent kinase 20 was amplified by PCR with primers appending XhoI and HindIII restriction sites, digested with XhoI and Hind III, gel isolated, and ligated (T4 DNA Ligase; NEB) in frame with a gene expressing green fluorescent protein (GFP) in the plasmid phCMV‐CGFP (Genlantis). Following transformation into chemically competent *E. coli* (NEB), single colonies were selected for growth, plasmid DNA isolation, and characterization by Sanger sequencing (Genewiz, Inc.).

### Primer design

*I. Vector Primer Design*. Primers were designed for the amplification of the target plasmid with the forward primer annealing to the 17‐bp region corresponding to the N terminus of GFP and the reverse primer annealing to the 17‐bp region corresponding to the C terminus of PKAc. *II. Library Primer Design*. Primers for the creation of linker libraries were designed with repeat sequence motifs, so that corresponding forward primers have multiple annealing sites with a given reverse primer. Primer sequences were designed to preclude potential frameshifts. For example, the repeat sequence encoding a poly‐Lysine linker utilized aag codon repeats rather than aaa codon repeats (Table 4). Both forward and reverse library primers have 17 bp of homology with appropriate regions of the target plasmid. Oligomers were obtained from Integrated DNA Technologies (Coralville, IA).

### PCR protocol

Forward and reverse primers for linker library generation were combined in a 1 : 1 ratio (12.5 μL of a 20 mm stock solution per primer) in 50 μL PCRs (25 μL of 2X Q5 master mix (New England Biosciences)) and cycled according to manufacturer's instructions for 35 cycles with T_m_ set according to calculated primer melting temperatures and an extension time of 30 s. For the GGS, GGSD, poly G, and poly KR linker series, *T*_m_ = 72 °C. For the poly K and poly L linker series, *T*_m_ = 55 °C. Linker library PCRs were utilized in downstream cloning steps without additional purification. Target vector amplicons were created using forward and reverse vector primers as described in Primer Design and amplified with Q5 PCR polymerase (NEB). Vector PCR amplicons were digested with DpnI for 1 h at 37 °C followed by gel isolation and extraction (see Results & Discussion).

### Fast cloning reaction

2 μL of the linker library PCR was combined with 2 μL of the vector PCR product (20 ng·μL^−1^) and incubated for 20 min at room temperature. 2 μL of the resulting reaction was transformed into 50 μL of XL10‐Gold Ultracompetent cells (Stratagene) with BME, following manufacturer's instructions. After 30 min of incubation on ice, transformation reactions were heat‐shocked at 42 °C for 30 s and then incubated for 1 h at 37 °C in 300 μL of SOC media with shaking. After 1 h, the entire transformation mixture (350 μL) was plated onto LB agar plates (Kanamycin) and allowed to incubate overnight at 37 °C. Following transformation into chemically competent *E. coli* (NEB), single colonies were selected for growth, plasmid DNA isolation, and characterization by Sanger sequencing.

### Cell culture, transfection, and lysis

HeLa cells were maintained in DMEM/10% FBS/1% Pen‐Strep antibiotic (Gibco) in a 37 °C/5% CO_2_/humidified tissue culture incubator (VWR). Two days prior to transfection, cells were plated at a density of 200 000 cells per well of 6‐well tissue culture plates. 1 μg of DNA was combined with Lipofectamine 3000 reagent (Invitrogen/ThermoFisher Scientific) following manufacturer's instructions. Briefly, 5 μL of P3000 reagent and 1 μg total plasmid DNA were combined in 125 μL of Opti‐MEM. At the same time, 3.75 μL of Lipofectamine 3000 reagent was added to 125 μL of Opti‐MEM. The two solutions were combined and incubated at for 10 min at room temperature. 250 μL of the resulting solution was added dropwise per well of a 6‐well tissue culture plate. One day post‐transfection, cells were harvested by removing cell media via aspiration, rinsing quickly with 1 mL of DPBS (supplemented with Ca^2+^ and Mg^2+^), and incubating with 300 μL of M‐PER lysis buffer (Thermo Fisher Scientific) with HALT protease and phosphatase inhibitor (10 min with shaking). Lysates were spun at 4 deg for 15 min in refrigerated centrifuge (Eppendorf 5424R; 18 407 ***g***; rotor FA45‐24‐11), and resulting supernatants were harvested for analysis. Total protein concentration was determined by Bradford assay (ThermoFisher Scientific). Lysates were combined with 6X Laemmli sample buffer and heated at 65 °C for 10 min prior to electrophoresis. Prior to lysis, control cells were treated with 70 μm forskolin (from a 10 mm DMSO stock solution) for 10 minutes at 37 °C. Control cells not treated with forskolin received an equivalent volume of DMSO.

### Western blotting

Protein lysates were loaded in equal amounts (20 μg total protein/lane) and subjected to SDS/PAGE using 10% Bis‐acrylamide protein gels in TGS running buffer. Proteins were transferred to PVDF membrane (20V/overnight) and subsequently blocked for 1 h in 5% BSA‐TBST. Blots were then incubated with primary antibody overnight at 4 °C, washed 3 × 5 min with TBST, and incubated with secondary antibody for 1 h, followed by washing 3 × 5 min with TBST, and rinsing with ddH2O prior to incubation with chemiluminescent substrate. 34 Primary antibodies were used at the following dilutions: α‐GFP (1 : 1000; Santa Cruz Biotechnologies sc‐8334); α‐phospho PKA substrate (RRXS*/T*) (1 : 1000; Cell Signaling Technologies 9624); α‐phospho VASP (Ser 157) (1 : 200; Santa Cruz Biotechnologies 23506); and α‐GAPDH (1 : 1000; ThermoFisher PIMA515738).

### Immunostaining and microscopy

Cells were plated and transfected as previously described in 35‐mm Mattek dishes. Prior to fixation, cells were washed with 1 mL of DPBS (Gibco) followed by the addition of 2 mL of 4% PFA in DPBS. After 10 min of incubation at room temperature, the fixative solution was removed by aspiration, and the cells were washed with DPBS (2 × 1 mL). A blocking and permeabilization solution (antibody dilution buffer—Cell Signaling Technology) was applied for 30 min at room temperature, and then, the primary antibody (Phospho‐CREB/ATF1 (Ser133, Ser63); ThermoFisher MA1‐114) diluted 1 : 200 in CST Ab dilution buffer) was applied. Cells were incubated in primary antibody at 4 °C overnight, followed by washing with DPBS (3 × 5 min), and incubation with secondary antibody (Texas Red‐X (Invitrogen T6391); 1 : 500) for 1 h at room temperature. Cells were subsequently washed with DPBS (3 × 5 min), stained briefly with Hoechst 33342, and washed again. Images were acquired with an Olympus IX2‐DSU tandem spinning‐disk confocal compound light microscope utilizing a 60x oil immersion objective. Nuclear fluorescence intensities were measured in FIJI/ImageJ 35 and quantified by the corrected total cell fluorescence method (CTCF) 36. Briefly, Individual cells were outlined in FIJI followed by calculation of area, integrated density, and the mean gray value for each cell. For each image, background measurements of fluorescence intensity were taken from regions without cells. The following formula was then used to calculate the CTCF for each cell: CTCF = Integrated Density – (Cell Area * Mean Background Fluorescence). CTCF values were averaged for 10 cells per experimental condition.

### Structure calculations

Structure calculations were carried out via the PEPFOLD 2.0 *de novo* peptide structure prediction server 28, 29.

## Acknowledgements

This work was supported by start‐up funds from the East Carolina University Office of Research, Economic Development, and Engagement, the Thomas Harriot College of Arts and Sciences, and the ECU Department of Chemistry.

## Author contributions

Experimental design (RMH); conducted experiments (JLN; RMH); wrote the manuscript (JLN; RMH).

## Supporting information


**Data S1**. The nucleotide sequence of the PKAc‐GFP fusion protein.
