# Supplementary material for: protaTETHER – a method for the incorporation of variable linkers in protein fusions reveals impacts of linker flexibility in a PKAc‐GFP fusion protein
Source: FEBS Open Bio. 2018 Apr 25;8(6):1029–42. doi: 10.1002/2211-5463.12414 (PMC5986021; doi:10.1002/2211-5463.12414)
Supplement: Supplementary file 2 [file FEB4-8-1029-s002.docx]

**Data S1**. The nucleotide sequence of the PKAc-GFP fusion protein.
